# Unraveling the Structural Changes in the DNA-Binding Region of Tumor Protein p53 (*TP53*) upon Hotspot Mutation p53 Arg248 by Comparative Computational Approach

**DOI:** 10.3390/ijms232415499

**Published:** 2022-12-07

**Authors:** Ambritha Balasundaram, C. George Priya Doss

**Affiliations:** Laboratory of Integrative Genomics, Department of Integrative Biology, School of BioSciences and Technology, Vellore Institute of Technology, Vellore 632014, Tamil Nadu, India

**Keywords:** *p53*, R248, human cancer, pathogenicity, *p53*–DNA interaction, molecular dynamics simulation

## Abstract

The vital tissue homeostasis regulator p53 forms a tetramer when it binds to DNA and regulates the genes that mediate essential biological processes such as cell-cycle arrest, senescence, DNA repair, and apoptosis. Missense mutations in the core DNA-binding domain (109–292) simultaneously cause the loss of *p53* tumor suppressor function and accumulation of the mutant p53 proteins that are carcinogenic. The most common *p53* hotspot mutation at codon 248 in the DNA-binding region, where arginine (R) is substituted by tryptophan (W), glycine (G), leucine (L), proline (P), and glutamine (Q), is reported in various cancers. However, it is unclear how the *p53* Arg248 mutation with distinct amino acid substitution affects the structure, function, and DNA binding affinity. Here, we characterized the pathogenicity and protein stability of p53 hotspot mutations at codon 248 using computational tools PredictSNP, Align GVGD, HOPE, ConSurf, and iStable. We found R248W, R248G, and R248P mutations highly deleterious and destabilizing. Further, we subjected all five R248 mutant-p53–DNA and wt-p53–DNA complexes to molecular dynamics simulation to investigate the structural stability and DNA binding affinity. From the MD simulation analysis, we observed increased RMSD, RMSF, and Rg values and decreased protein–DNA intermolecular hydrogen bonds in the R248-p53–DNA than the wt-p53–DNA complexes. Likewise, due to high SASA values, we observed the shrinkage of proteins in R248W, R248G, and R248P mutant-p53–DNA complexes. Compared to other mutant p53–DNA complexes, the R248W, R248G, and R248P mutant-p53–DNA complexes showed more structural alteration. MM-PBSA analysis showed decreased binding energies with DNA in all five R248-p53–DNA mutants than the wt-p53–DNA complexes. Henceforth, we conclude that the amino acid substitution of Arginine with the other five amino acids at codon 248 reduces the p53 protein’s affinity for DNA and may disrupt cell division, resulting in a gain of p53 function. The proposed study influences the development of rationally designed molecular-targeted treatments that improve *p53*-based therapeutic outcomes in cancer.

## 1. Introduction

*TP53* gene encodes a tumor-suppressor protein called *p53* (also known as the guardian of the genome), which causes apoptosis in response to oncogenic stress. p53 plays a significant role in cell division regulation and monitoring. Loss of function occurs due to mutations in the *p53* gene or abnormalities in the upstream or downstream *p53* signaling pathways that are vital for malignant development [1,2]. The short arm (p13) of chromosome 17 encompasses the *p53* gene. It has 11 exons spanning 20 kilobases and forms a 53-kDa core phosphoprotein. The 393 amino acid residue p53 protein comprises four functional domains: N-terminus transactivation (1–63) and proline-rich region (64–92), sequence-specific DNA binding (102–292), oligomerization (323–356), and the C-terminus negative regulatory (356–393) domain [3,4]. p53 can facilitate some of its functions independently of transactivation. Despite this, its induction includes direct or indirect activation of multiple genes that regulate growth factors, apoptosis, suppression of genes involved in cell metabolism, growth arrest, and the cytoskeleton [5,6].

*p53* gene mutations are reported in 50% of all human malignancies [3,7]. Mutated *p53* genes are found in 96% of cases of serous ovarian carcinoma, 85% of cases of small cell lung cancer, 75% of cases of pancreatic cancer, 60% of cases of head and neck squamous cell carcinoma, and 54% of cases of invasive breast carcinoma [8]. *p53* gene mutations are also identified in 35–60% of NSCLC patients with squamous cell carcinomas and people with a more common smoking history (particularly G > T transversions) [9]. Approximately 5% of *p53* somatic mutations are reported in leukemia, sarcoma, malignant melanoma, cervical cancer, and testicular cancer. Mutation rates are more significant in advanced stages/advanced cancer subtypes, such as triple-negative breast cancer [7,10]. Nearly 80% of the mutations in the *p53* gene are missense mutations [11]. Most missense mutations are found in the DNA-binding domain, resulting in the loss of target gene transactivation [12]. Mutations found in the DBD are also known as hotspot mutations. Six p53 protein residues in DBD, namely Arg175, Gly245, Arg249, Arg248, Arg273, and Arg282, are often altered in human cancer [13,14]. These six residues account for 30% of *p53* gene mutations in all human cancer. According to the COSMIC database (https://cancer.sanger.ac.uk/cosmic, accessed on 30 April 2022), Arg248 is the second most prevalent mutation in the p53 DBD region [15]. The missense mutations found in the DNA-binding region of the p53 protein fall into categories: directly interfere with p53–DNA interaction by contact mutations (R248Q and R273H) and indirectly affect p53–DNA interaction by causing local (R249S and G245S) or global (R175H and R249W) conformational changes [16,17,18]. R248 and R273 are two major DNA-binding sites with the highest cancer mutation frequencies [7,19]. The p53 R248 hotspot is perhaps the most prevalent mutation across all *p53*-modified tumor types, accounting for 9% of instances, and approximately 66,000 recently diagnosed cancer patients carry R248 mutations in the US annually [13,20]. Every mutation has a unique set of traits. The distinct amino acid changes at the exact location might have different functions. In R248Q, mutations in lung cancer cell lines increase in vitro invasiveness, whereas R248W mutations in human NCI-H1299 cell lines do not increase invasiveness. [21]. Similarly, R273H and R273C mutations in cell lines promote drug resistance, cell proliferation, and invasion, whereas R273G does not [22]. R248W mutants are linked with increased mortality rates in breast cancer [23]. Patients with high-grade serous ovarian cancer who carry the p53 R248 mutation present worse survival rates. Further, it was observed that R248 mutated ovarian cancer cells are mainly resistant to chemotherapy drugs and taxane [24]. Anti-cancer medicines that directly target mutp53 are indeed a long way off the tiny chemical APR-246 (PRIMA-1MET), which restores mutp53 to a wild-type state, and is the only one that has made it to Phase I/II clinical trials. These might become the therapies of choice for cancers with an increased *p53* mutation rate, but lack focused, effective therapy alternatives [25]. Even though missense mutations have different functional effects, tumor vulnerabilities might vary depending on the *p53* mutation and tumor type [26].

The current study focuses on the DNA-contact R248 position of the *p53* gene, which has more excellent mutation rates than other DNA-contact mutations and has several oncogenic mutations in different cancers. We assessed the pathogenicity of all five mutations, R248W, R248G, R248L, R248P, and R248Q, found in the R248 position of the p53 protein using computational tools, PredictSNP [27], Align GVGD [28], HOPE [29], ConSurf [30], and iStable [31] and further investigated the structural behaviors and the binding affinity of DNA with p53 protein via molecular dynamics (MD) simulations and analysis using GROMACS [32]. This proposed research will assist in better understanding the structural behavior and DNA binding affinity of the DNA-contact R248 mutations with different amino acid substitutions seen in human cancers. Furthermore, this research will aid in developing residue-specific treatment techniques for cancer.

## 2. Result

### 2.1. The p53 Missense Mutations of the Residue R248

Based on the COSMIC database, 2090 counts of R248 mutations reported *p53* with different amino acid substitutions in different tumor types of cancers. The residue arginine (R) at the 248 position was substituted with tryptophan (W), glycine (G), leucine (L), proline (P), and glutamine (Q) in the *p53* gene. The frequency of R248Q 1541 (52%), R248W 1211 (41%), R248L 157 (5%), R248P 40 (1%), and R248G 34 (1%) mutations of the *p53* gene in different cancers was assessed. We subsequently analyzed all five mutations to distinguish their pathogenicity and stability.

### 2.2. Detection of Deleterious Missense Mutations Using Computational Approaches

The five missense mutations at the R248 position were examined using PredictSNP, an online SNP analyzing tool package system that includes diverse SNP analyzing methods. It utilized the p53 protein sequence and missense mutations position as input and generated the different values and percentages for each missense mutation. The PredictSNP tool predicted that all the five missense mutations at R248 were deleterious in MAPP [33], PolyPhen-1 [34], PhD-SNP [35], SIFT [36], and SNAP [37] tools, whereas nsSNPAnalyzer [38], PANTHER [39], and PolyPhen-2 [40] predicted the R248 missense mutations as unknown (Table 1).

In addition, Align Grantham Variation Grantham Deviation (Align GVGD) techniques were utilized in conjunction with MSA to categorize the variant scores into C0, C15, C25, C35, C45, C55, and C65 [41]. The score of C0 is denoted as least likely to be deleterious and C65 as most likely to be deleterious. We found that R248W, R248G, R248P, and R248L mutations belong to the C65 class, indicating that they are more likely to cause damage. Using the ConSurf tool, we analyzed the evolutionary conservation region of the R248 position in the p53 protein and found the highest conservation score of nine, which indicates that the R248 position is an evolutionarily conserved region of the p53 protein (Table 2). iStable identified four out of five missense mutations at R248 that decreased p53 protein stability in all three predictors, whereas R248L mutation predicted increased p53 protein stability by MUpro [42] and iStable tools and decreased p53 protein stability by I-Mutant2.0 SEQ [43] (Table 2).

### 2.3. Structural Changes of R248 Missense Mutations in p53 Protein Using HOPE

We evaluated the mutant proteins’ three-dimensional (3D) structure using the HOPE server. The 3D structure reveals the structural insights contributing to the disease. The amount of mutant R248G, R248P, R248L, and R248Q residue was smaller than the wild-type residue, whereas the mutant R248W residue was more abundant than the wild-type residue. The mutant residues R248W, R248G, R248L, and R248P, were more hydrophobic than the wild-type residue. The wild-type residue charge was positive, whereas the mutant R196P residue charge was neutral. The mutations R248G, R248L, and R24P, were annotated as severe. These mutations will result in a lack of interactions with the DNA, which will disrupt cell division. From this, altered size, charge, hydrophilicity, and flexibility of the R248 mutation significantly affect the structural disruption of the protein and the binding affinity with DNA, resulting in altered function.

### 2.4. Molecular Dynamics Simulations

**Figure 1:**
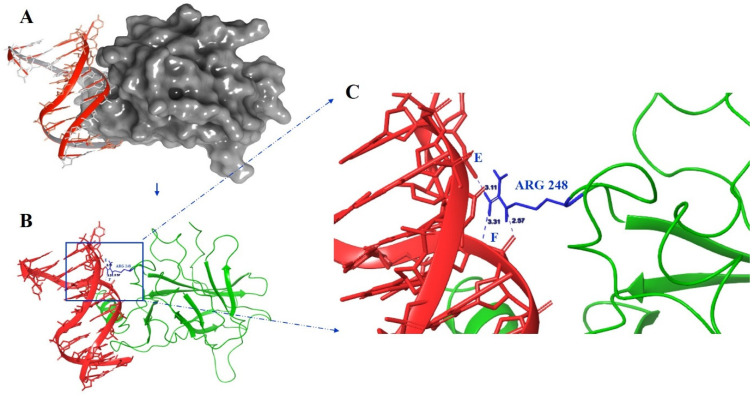
(**A**) The p53–DNA complex structure was obtained from the Maestro suite. The DNA is represented in the red cartoon, and their atoms are shown as red stick models, whereas the protein surface is represented in the grey surface model. (**B**) The 3D structure of the p53 protein (PDB ID-2AC0) shows the p53 protein (labeled in green color) interacting with DNA (labeled in red). Blue box highlighted the three H-bonds with wild-type Arg-248 residue (marked in blue) and their distance are blue labeled in Å. (**C**) Interaction of DNA with R248 residue of p53. DNA chain E of 2AC0 structure formed a hydrogen bond with chain A of R248 residue of p53 protein with (A: Arg248:NH2-E:Gua12:O2P). Similarly, DNA chain F of 2AC0 structure formed two H-bonds with chain A of R248 residue of p53 protein (A:Arg248:NH2-FAde6:O3′ and A:Arg248:NE-F:Thy7:O1P).

We subjected 100ns MD simulations towards wt-p53–DNA (PDB ID:2AC0) [44] and mutant R248W-p53–DNA, R248G-p53–DNA, R248Q-p53–DNA, R248P-p53–DNA, and R248L-p53–DNA complexes to comprehend their binding affinity with the DNA. We explored the root mean square deviation (RMSD), the root mean square fluctuation (RMSF), the number of hydrogen bonds (H-bonds), the radius of gyration (Rg), the solvent accessible surface area (SASA), the molecular mechanics Poisson–Boltzmann surface area (MM-PBSA), and the principal component analysis (PCA) between DNA and protein complexes. We illustrated the 3D p53–DNA complex structure (PDB ID-2AC0) and marked the residue R248 interacted regions with DNA using Maestro (Figure 1).

**Figure 2:**
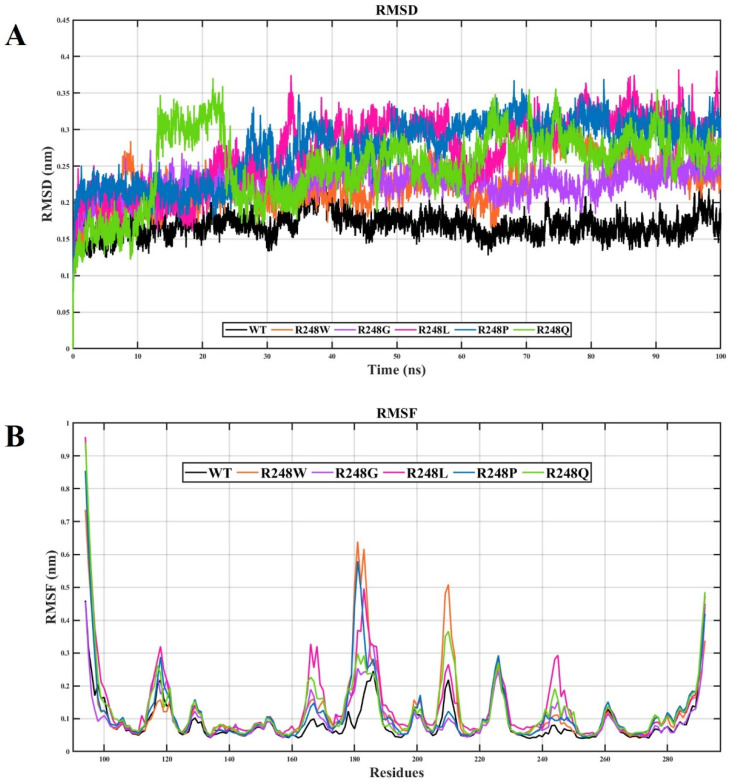
(**A**) RMSD plot of the wt-p53–DNA complex and R248 mutant-p53–DNA complexes. The X and Y axes denote time (ns) and RMSD (nm), respectively. (**B**) RMSF plot of the wt-p53–DNA complex and R248 mutant-p53–DNA complexes. The X and Y axes denote residues and RMSF (nm), respectively. Color representation is as follows: black (Wt-p53–DNA complex), orange (R248W mutant-p53–DNA complex), violet (R248G mutant-p53–DNA complex), pink (R248L mutant-p53–DNA complex), blue (R248P mutant-p53–DNA complex), and green (mutant-p53–DNA complex), respectively.

The RMSD of the backbone in the initial structure was investigated to measure the convergence pattern of the p53 protein system. In the RMSD plot, the mutant-p53–DNA complexes (R248W, R248G, R248Q, R248P, and R248L) exhibited a higher deviation pattern than the wt-p53–DNA complex (Figure 2A). The RMSD curve illustrates the p53 protein backbone stability change in response to five different amino acid substitutions at position R248. The wt-p53–DNA complex showed the most negligible RMSD value of ~0.15 throughout the 100 ns. RMSD values for mutant R248W and R248G mutant-p53–DNA complexes range from ~0.15 to ~0.25, for R248L mutant-p53–DNA complex range from ~0.2 to ~0.35, for the R248P mutant-p53–DNA complex range from ~0.2 to ~0.32, and for the R248Q mutant-p53–DNA complex range from ~0.2 to ~0.35, respectively. The R248Q mutant-p53–DNA complex stabilized around ~0.1 to ~0.2nm. After 12ns onwards, the RMSD spiked to ~0.35 until 22ns, maybe due to some structural alteration. As mentioned above, the obtained RMSD values were higher in mutant-p53–DNA complexes than in the wt-p53–DNA complex. A higher RMSD value primarily indicates a trajectory that deviates from the wt-p53–DNA complex with the R248 mutant-p53–DNA complex. This indicates that a substantial conformational alteration in the p53 protein–DNA complex may occur with the R248 mutation. After 70 ns, the mutant-p53–DNA complex stabilized with minimal drifts. Further, the RMSF of C α-atoms of all amino acids was analyzed to study the wt-p53–DNA complex and mutant-p53–DNA complex’s dynamic behavior, as depicted in Figure 2B. The residues ranging from ~165 to ~172, ~180 to ~188, and ~240 to 250 showed more fluctuations in mutant-p53–DNA complexes than the wt-p53–DNA complex. Mutant-p53–DNA complexes (R248W, R248L, and R248Q) also showed higher fluctuations than the wt-p53–DNA complex between ~208 to ~215 residues compared to R248G and R248P mutant-p53–DNA complexes. Overall, RMSFs of R248W, R248L, R248Q, R248G, and R248P mutant-p53–DNA complexes deviated between the residues compared to the wt-p53–DNA complex structure. The difference mentioned above in RMSF values might indicate changes in the internal dynamics and binding intensities with DNA. As a result, the stability of an individual residue during MD simulation may be affected by interactions with the original residue R248 that mutates to different amino acids.

**Figure 3:**
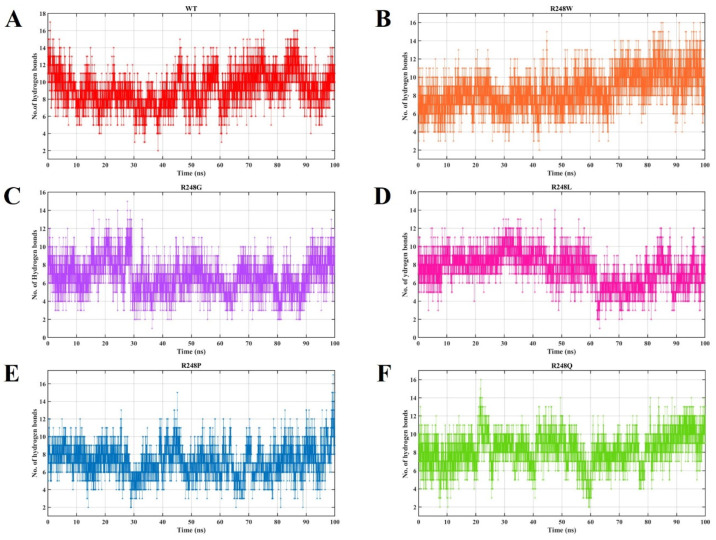
Number of H-bonds between the p53 protein and DNA in the wt-p53–DNA complex and R248 mutant-p53–DNA complexes. The X and Y axes denote time (ns) and the number of H-bonds. (**A**) The red color represents the wt-p53–DNA complex, (**B**) the orange color represents the R248W mutant-p53–DNA complex, (**C**) the violet color represents the mutant R248G mutant-p53–DNA complex, (**D**) the pink color represents the mutant R248L mutant-p53–DNA complex, (**E**) the blue color represents the mutant R248P mutant-p53–DNA complex, and (**F**) the green color represents the mutant R248Q mutant-p53–DNA complex.

The number of intermolecular H-bonds formed within the p53 protein and DNA in the wt-p53–DNA and R248 mutant-p53–DNA complexes (R248W, R248G, R248Q, R248P, and R248L) (Figure 3) measures the observed changes in RMSD. The average number of H-bonds in the wt-p53–DNA complex is 9.21 (range from 2 and 17). The average number of H-bonds in mutant-p53–DNA complexes R248G (ranges from 1 to 15), R248L (ranges from 1 to 14), R248P (range from 2 to 17), R248W (ranges from 2 to 16), and R248Q (ranges from 2 to 16) were identified as ~6.77, ~7.53, ~7.23, ~8.54, and ~8.30, respectively. The mutant-p53–DNA complexes had fewer H-bonds than the wt-p53–DNA complex, resulting in a more divergent pattern in the RMSD analysis. The more significant deviation elucidates the damaging effect of forming fewer hydrogen bonds between the protein and DNA.

**Figure 4:**
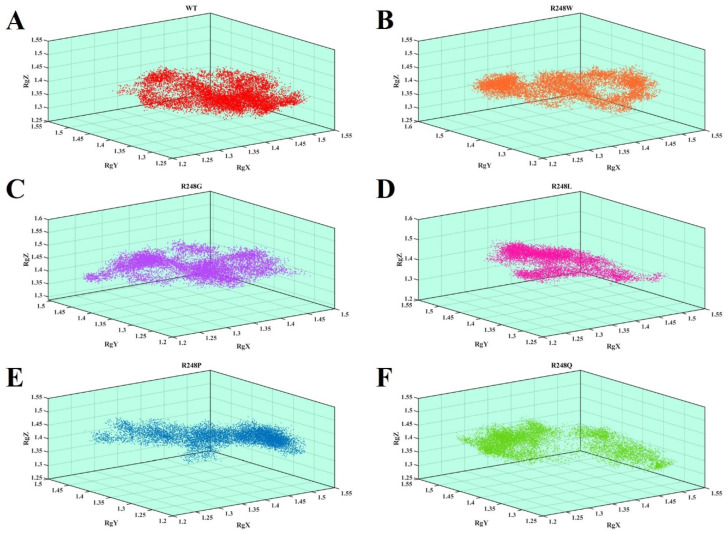
The 3D scatter radius of gyration (Rg) plot of the wt-p53–DNA and R248 mutant-p53–DNA complexes. The X and Y axes denote time (ns) and the number of Rg (nm). (**A**) The red color represents the wt-p53–DNA complex, (**B**) the orange color represents the R248W mutant-p53–DNA complex, (**C**) the violet color represents the R248G mutant-p53–DNA complex, (**D**) the pink color represents the R248L mutant-p53–DNA complex, (**E**) the blue color represents the R248P mutant-p53–DNA complex, and (**F**) the green color represents the mutant R248Q mutant-p53–DNA complex.

We used Rg to quantify and characterize the compactness of wt-p53–DNA and mutant-p53–DNA complexes. The wt-p53–DNA complex had an average Rg value of ~1.6939 during the 100 ns simulation. The average Rg values for mutant R248W, R248G, R248L, R248P, and R248Q mutant-p53–DNA complexes were ~1.6974, ~1.6975, ~1.7102, ~1.6982, and ~1.6993, respectively. The mutant-p53–DNA complexes obtained slightly increased Rg values compared to the wt-p53–DNA complex. An increased Rg value in mutant-p53–DNA complexes implies a decrease in complex structure compactness, suggesting increased flexibility and less stability in mutants than in the wt-p53–DNA complex. Further, the arrangement of structural conformations in the 3D image demonstrates that mutations of the p53 protein have lost the compactness of the p53–DNA structure (Figure 4).

**Figure 5:**
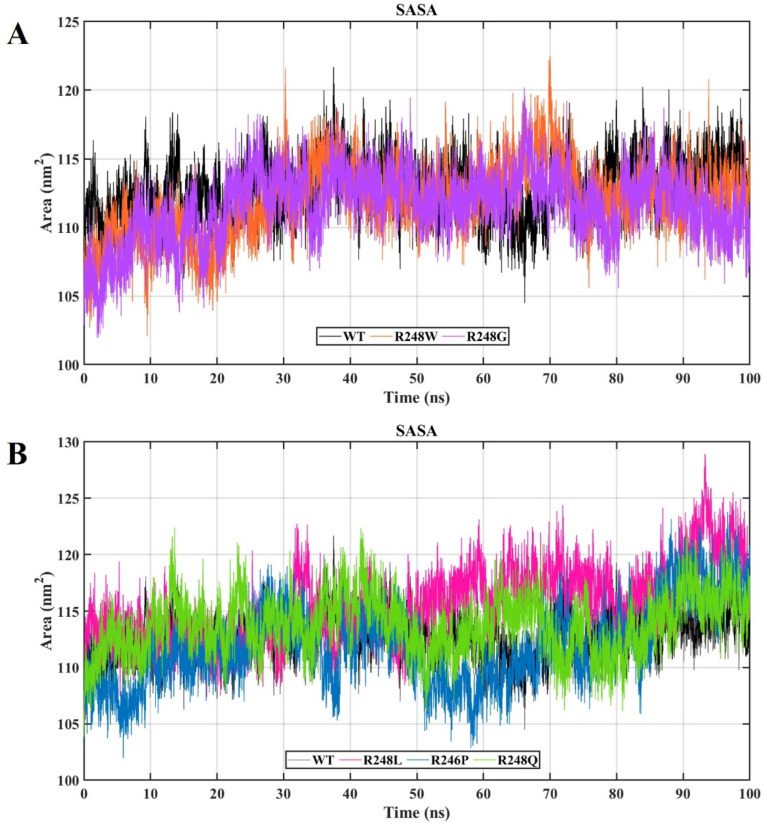
The SASA plot of the wt-p53–DNA and R248 mutant-p53–DNA complexes. The X and Y axes denote time (ns) and the area (nm^2^). (**A**) The black color represents the wt-p53–DNA complex, the orange color represents the R248W mutant-p53–DNA complex, and the violet color represents the R248G mutant-p53–DNA complex. (**B**) The black color represents the wt-p53–DNA complex, the pink color represents the R248L mutant-p53–DNA complex, the blue color represents the R248P mutant-p53–DNA complex, and the green color represents the R248Q mutant-p53–DNA complex.

Figure 5 depicts the change of SASA between wt-p53–DNA and mutant-p53–DNA complexes (R248W, R248G, R248L, R248P, and R248Q) throughout time (100 ns). The average SASA value was ~112.8278 in the wt-p53–DNA complex, whereas the R248W, R248G, R248L, R248P, and R248Q mutant-p53–DNA complexes had average SASA values of ~111.9959, ~111.5791, 115.5683, ~111.9909, and ~113.7803, respectively. The lower value of SASA in the mutant R248W, R248G, and R248P mutant-p53–DNA complex indicate that the structure was distorted compared to the wt-p53–DNA complex. The higher value of SASA in the R248L and R248Q mutant-p53–DNA complexes indicates an enlarged structure compared to the wt-p53–DNA complex. An increase or reduction in a protein’s SASA implies a shift in surface amino acid residues that may influence the protein’s tertiary structure. The structural rearrangement should account for the unique physiochemical features of the altered residue. Ultimately, MD simulation analysis indicated the differences in protein stability and fluctuation upon missense mutation.

**Figure 6:**
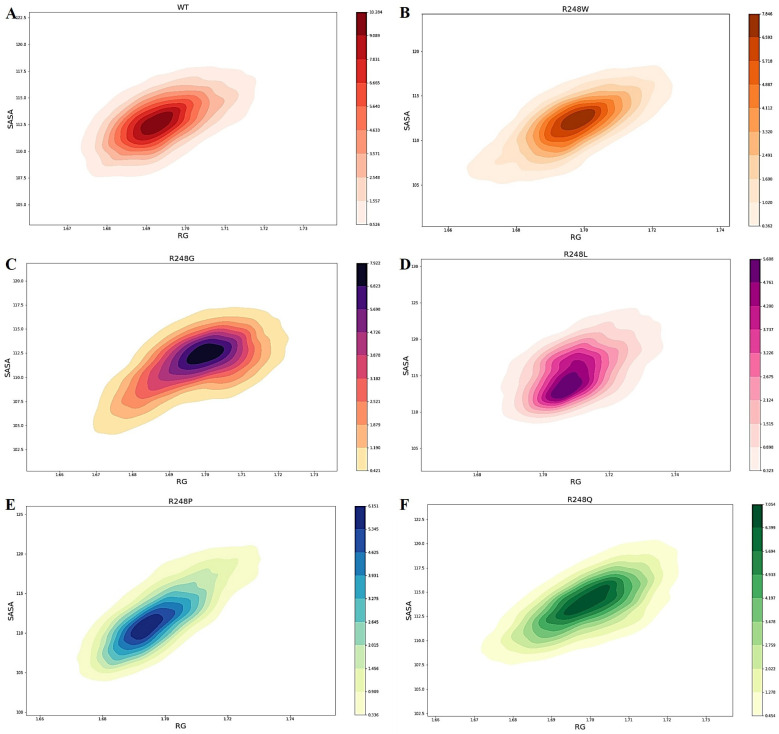
The kernel density estimation (KDE) plots of Rg and SASA were used as collective variables to visualize the conformation of the wt-p53–DNA complex and R248 mutant-p53–DNA complexes during the MD simulation. Rg (nm) is shown on the X-axis, while SASA (nm^2^) is depicted on the Y-axis. (**A**) The wt-p53–DNA complex, (**B**) the R248W mutant-p53–DNA complex, (**C**) the R248G mutant-p53–DNA complex, (**D**) the R248L mutant-p53–DNA complex, (**E**) the R248P mutant-p53–DNA complex, and (**F**) the R248Q mutant-p53–DNA complex.

Comparing Rg vs. SASA plots for wt-p53–DNA and R248 mutant-p53–DNA complexes quantifies two crucial global properties: total size and solvent exposure. We observed a drastic change in the overall size and solvent exposure of the mutant-p53–DNA complexes compated to the wt-p53–DNA complex, which is illustrated in Figure 6.

**Figure 7:**
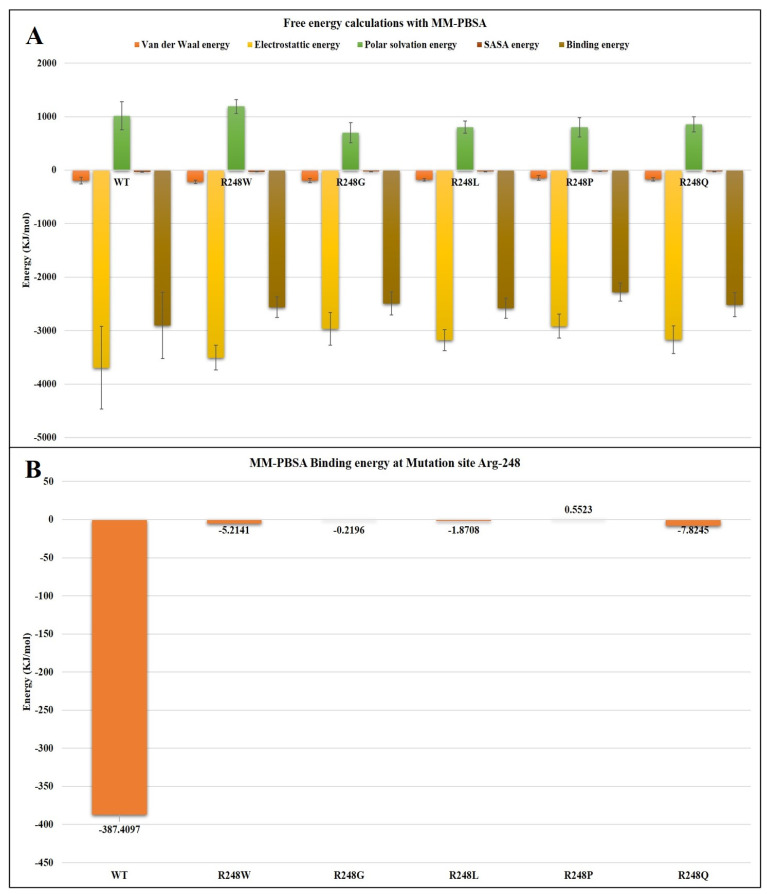
(**A**) Binding free energy with MM-PBSA calculation concerning the overall binding of DNA with wt-p53 and five different R248 mutant-p53 proteins as indicated in the decomposition plot, with a different color for each schematic representation of energy components. (**B**) MM-PBSA decomposition of binding free energy at mutation site Arg-248 was plotted on a per-residue basis with the contributions from electrostatic interactions, vdW energy, polar solvation energy, and SASA energy.

A comprehensive analysis of binding free energy was carried out using MM-PBSA to comprehend the molecular interaction and stability connected with the binding of DNA to the mutated R248 p53 proteins. MM-BPSA offers the most robust predictive performance for the energy factors of bonded, non-polar, and polar solvation-free energy, van der Waals, and electrostatic interactions. It also includes a residue-wise plot. We used this plot to investigate the involvement of mutated amino acid residue associated with the spatial interaction to stabilize DNA at the binding region of the protein. The last 50 ns of the completely converged trajectory were used for this investigation. The results indicate that p53 protein bonds to the DNA were unfavorable upon R248 mutations. The results also exhibit the smallest range of total binding free energies, which are listed in Table 3. According to Figure 7A, DNA binding affinity was lowest for R248 mutant-p53–DNA than for the wt-p53–DNA complexes. The wt p53 protein’s highest binding energy with DNA was −2906.756 ± 621.185 KJ/mol. The R248P mutant-p53–DNA complex obtained the lowest binding energy with DNA, −2283.598 ± 169.333 KJ/mol, compared to other R248 mutant-p53–DNA complexes. The binding energy for the R248G, R248W, R248Q, and R248L mutant-p53–DNA complexes are as follows: −2492.508 ± 215.762 KJ/mol, −2564.871 ± 194.196 KJ/mol, −2516.614 ± 227.002 KJ/mol, and 2583.670 ± 186.701 KJ/mol. The findings of the MD trajectory analysis and MM-PBSA indicated that the mutant p53 complex with DNA demonstrated the least binding energy on the mutation site Arg 248 residue based on the residue-wise analysis plot, as seen in Figure 7B. The binding energy at the mutation site Arg248 depends upon the number and type of H-bond formation between the p53 protein and DNA, as mentioned in Table 4.

**Figure 8:**
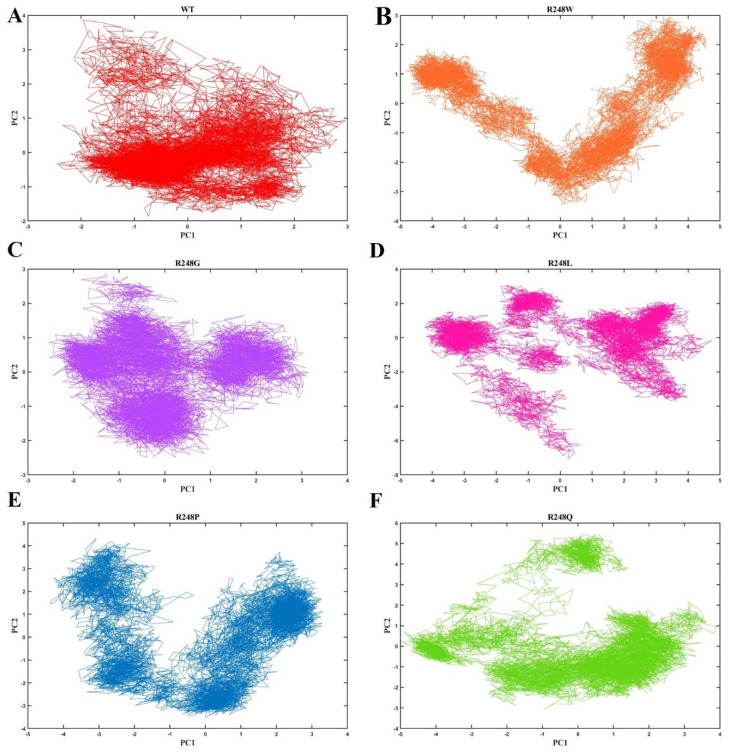
The principal component analysis (PCA) of the wt-p53–DNA complex and R248 mutant-p53–DNA complexes. (**A**) The red color represents the wt-p53–DNA complex, (**B**) the orange color represents the R248W mutant-p53–DNA complex, (**C**) the violet color represents the R248G mutant-p53–DNA complex, (**D**) the pink color represents the R248L mutant-p53–DNA complex, (**E**) the blue color represents the R248P mutant-p53–DNA complex, and (**F**) the green color represents the R248Q mutant-p53–DNA complex.

Protein activity was controlled by changing between different conformations. The collective motion of proteins, inherent to many biological processes and essential in transmitting signals, controls the flexible nature of proteins to flip among different states. Particularly for the residues in the binding region, a protein must be flexible and stiff to function. Generally, a tighter connection would limit the mobility of the protein, making it unable to choose some configurations necessary for its function. Hence, we used the feature reduction approach, essential dynamics (ED) evaluation through the projection of the first two principal components (PCs), PC1, and PC2, to determine the combined motion of proteins formed in the conformational space throughout the simulation. The fundamental subspace where most of the protein dynamics take place was defined by diagonalizing the eigenvector data matrix, from which the PC1 and PC2 were derived. Figure 8 depicts the dynamic mobility of the wt-p53–DNA and R248 mutant-p53–DNA complexes as determined by the projection of PC1 and PC2. The results obtained from the all-system simulations throughout the time demonstrate unequivocally that all mutant-p53–DNA complexes engaged a larger phase space area than the wt-p53–DNA complex, which covered a smaller phase space area. These findings suggested that R248 mutations in the p53 protein–DNA complexes induced a different conformational fluctuation in contrast to the wt-p53–DNA complex. Therefore, according to the PCA results, the wt-p53–DNA complex is more stable than the five mutant-p53–DNA complexes, and these R248 mutations in the p53 protein significantly changed the stability and flexibility of the structure. As a result, the analysis is more valid since the PCA results concur with the RMSD, RMSF, Rg, and SASA results (Figure 8).

## 3. Discussion

The p53 protein is a crucial regulator of cell proliferation or survival in reaction to diverse stressors, functioning as a fundamental anti-cancer defensive mechanism [45,46,47]. According to the TCGA cohort, *p53* is a frequent tumor suppressor mutation in NSCLC, accounting for 46% of mutations in lung adenocarcinoma [48]. *p53* mutations are common in many forms of cancer, resulting in the production of mutant p53 proteins. Extensive evidence from in vitro and cell culture experiments revealed that few missense mutations in p53 inhibit wt-p53 in a dominant-negative manner and result in the inactivation of cellular p53 function [19,49]. Most p53 mutations are found in the central DNA-binding domain that inactivates the function of transcription factors. The DNA minor groove is directly contacted by R248 (L3 loop) in the p53 DNA binding domain (DBD). The dynamics of the L3 loop where the R248 mutation is situated rely on the charge and steric. Furthermore, it has been reported that the R248Q mutation in the p53 protein alters the global conformation [50].

**Figure 9:**
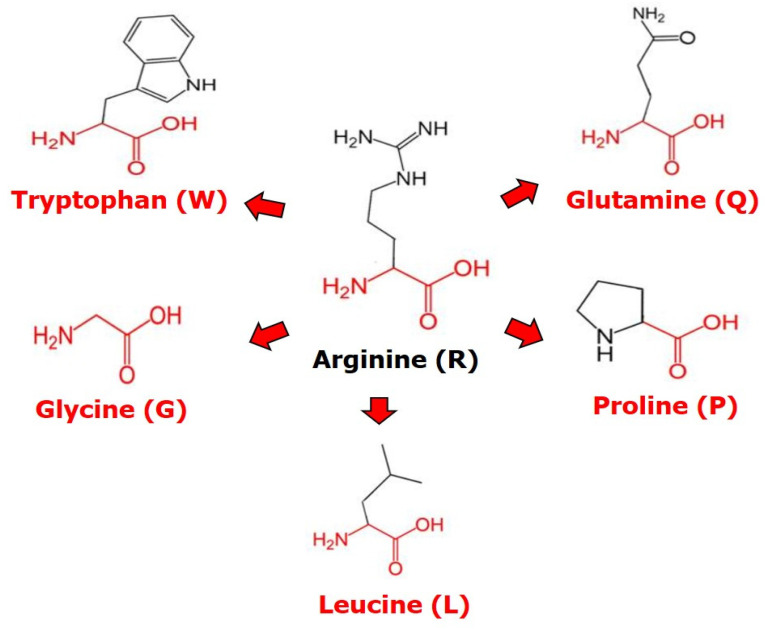
Structural relativity of different amino acid substitutions in p53 Arg248.

The proposed study aimed to explore the five different amino acid replacements present in the DNA-binding R248 position of the *p53* gene, and the dynamic behavior of the protein–DNA interactions was studied using MD simulations. We analyzed the pathogenicity of all five mutations at position 248 of the p53 protein using computational tools such as SIFT, PredictSNP, Align GVGD, iStable, and ConSurf. PredictSNP predicted that all the five missense mutations at R248 have deleterious effects on the p53 protein using MAPP, PolyPhen-1, PhD-SNP, SIFT, and SNAP tools. iStable predicted that R248W, R248G, R248Q, and R24PL missense mutations decrease p53 protein stability. Align GVGD predicted R248W, R248G, R248P, and R248L mutations to be in the C65 class, indicating that they are more likely to cause damage. Although R248Q belongs to class 35, it is still considered potentially harmful [41]. ConSurf revealed the highest conservation score of nine for the R248 position. By incorporating the results obtained from PredictSNP, iStable, Align GVGD, and ConSurf tools, we found R248W, R248G, and R248P mutants to be highly deleterious and destabilizing. Further, the structural changes of these proteins upon mutant R248W, R248G, R248L, R248P, and R248Q were studied using the HOPE server. We compared the structure relative to the arginine (R) residue substituted with five different amino acids using HOPE, as illustrated in Figure 9. Furthermore, we also performed an MD simulation to understand the binding affinity of p53–DNA interactions upon DNA R248 mutations. The p53 interaction with DNA binding regions was composed of two decameric motifs or primary half-sites type RRRCWWGYYY (R=A, G; W=A, T; Y=C, T) spacing of 0–13 bp in a sequence-specific way [51,52]. Since most of the missense mutations arise from the core domain, the significance of p53′s sequence-specific DNA binding region was emphasized due to its ability to function as a tumor suppressor protein. p53 creates tetramers, the protein’s basic functional unit, when it binds to DNA targets with two half-site motifs [53,54,55]. p53-dependent gene expression is represented in the different sequences of its specific DNA sites and their corresponding arrangements, according to research on DNA binding by p53 and transcriptional activation [56,57]. Hence, we utilized the monomer crystal structure of p53–DNA complexes from PDB ID 2AC0 to understand the functional behavior of the deleterious missense mutations at R248 [44]. Missense mutations, which arise in the protein’s DNA-binding regions, have previously been reported to inhibit protein activity and affect the DNA-binding affinity [58,59]. Protein stability and function are two interconnected aspects to be evaluated while investigating protein structure [60,61]. Any mutations that suppress a protein’s properties might directly affect the functions. Therefore, protein stability is vital for maintaining its function [62]. According to research on the evolution stability and mutational resistance in protein-coding genes, arginine, leucine, and serine are essential amino acids that impact protein stability among mutants [63]. The most frequently altered residue in human p53, Arg 248, plays a crucial part in DNA binding. DNA binding is decreased by Arg 248 and Arg 273 mutations because they break phosphate backbone linkages [64].

In the present study, we observed higher deviations for the mutant-p53–DNA complexes in RMSD and RMSF (Figure 2). H-bonds are the most significant interactions in biological recognition processes and play a crucial role in binding specificity [65]. Intermolecular H-bonding can provide significant binding affinity between the protein and DNA [66]. We observed fewer intermolecular H-bonds in the R248 mutant-p53–DNA complexes than in the wt-p53–DNA complex (Figure 3). p53 transcription factors are also involved in hydrogen bonding with the protein and DNA. Fewer intermolecular H-bonds in the R248 mutant-p53–DNA complexes lowers the affinity between protein and DNA, which may affect function [67]. We found that Rg was greater in the R248 mutant-p53–DNA complexes than in the wt-p53–DNA complex, signifying a decline in the compactness of the mutant- p53–DNA complexes (Figure 4). The above analyses show that the protein’s structural compactness reduces with a decrease in H-bonds [68].

Moreover, in SASA analysis, we observed the decreased SASA value for R248W, R248G, and R248P mutant-p53–DNA complexes compared to the wt-p53–DNA complex, suggesting that the mutation that occurred at the DNA-binding site results in the shrinkage of the p53 protein (Figure 5A,B). We calculated the overall binding free energies and mutation site residue binding free energies between the wt-p53–DNA complex and mutant-p53–DNA complexes. We observed lower binding energies for R248 mutant-p53–DNA complexes than the wt-p53–DNA complex (Figure 7A,B). Therefore, we found that R248 mutations in the p53 protein were structurally unstable due to lower binding energy, and this unstable nature disrupts the p53–DNA interaction pattern, resulting in a gain of p53 activity [19]. Based on the KDE plot of Rg and SASA, there is a change in the size and solubility nature of the R248 mutant-p53–DNA complexes compared to the wt-p53–DNA complex (Figure 6). PCA analysis allowed us to identify the functional movements of Cα atoms [69]. We observed different configurations for the R248 mutant-p53–DNA complexes compared to the wt-p53–DNA complex. This further demonstrates that mutation affects the functional movements of the protein (Figure 8). Previous data from NMR and in silico simulations have shed light on the L3 structural alterations with rearrangements of L2 brought on via the conversion of arginine to glutamine in the p53 R248Q contact mutant [50,70]. Our study reported that the R248Q mutant changed the binding affinity with DNA along with the structural alterations. The study compared SAS cells that expressed R248Q to the parent or mock-transfected cells and found that they exhibited highly mobile, invasive, and spreading behaviors in ovarian squamous cell carcinoma (OSCC) cells [71]. As an outcome, the structural changes and affinity of the R248Q mutant may contribute to invasive carcinoma. Even though structural information was lacking earlier, they hypothesized that tryptophan would displace arginine in the contact mutation p53 R248W, abolishing the harbor of the p53 to DNA minor grooves because tryptophan is hydrophobic and sterically clashes with arginine, preventing the formation of H-bonds with DNA [72]. Based on our computational approach, we can clearly understand that R248W mutant-p53 prevents the formation of H-bonds with DNA. Colorectal cancers with *p53* R248Q/W have a greater probability of patient mortality than cancers without this mutation [73]. Some particular mutations of mutp53, such as R175H, R248W, R273H, and R280K, have been closely linked to gain of function (GOF) [72]. In glioblastoma (GBM), differently expressed genes associated with chemotaxis and inflammation are upregulated by *p53* R248L [74].

In the past decade, the relevance of protein interfaces as centers for disease-associated mutations has become more well-recognized [75,76,77]. A mutation on the protein’s DNA interaction region can destabilize the interaction partners, disrupt the partner’s binding, enhance, diminish, or modify the partner’s affinity, or stabilize the interface. This relationship is strongly linked to disease states [78,79]. Experiments significantly support the concept that many pathogenic alleles disrupt protein interaction rather than destabilize protein structure [80]. Researchers have recently focused on MD simulation studies to elucidate the varied consequence of mutations present in the protein and interactions between the protein–ligand and protein–DNA [13,81,82]. MD simulations of the protein conformations associated with particular biological activities can aid in the discovery of conformationally selective ligands [83]. Furthermore, this effective strategy is strongly associated with experimental studies [84]. PhiKan083 binds to the surface cavity produced by the conformational mutation Y220C, which severely destabilizes the p53 protein. The crystal structure and NMR-based in silico screen of the ZINC database led to the discovery of the PhiKan083 [85]. Immunofluorescence measurements revealed that PK7088 boosted the quality of folded mutant protein with wild-type structure and restored its transcriptional capabilities. It caused apoptosis, cell-cycle arrest, and growth suppression dependent on p53 Y220C [86]. Structure-based drug design is a fast-expanding subject that had much success recently. Numerous novel targets and prospects for identifying new therapeutic leads have been made possible by the explosion of genomic, proteomic, and structural information [87].

Understanding the exact molecular structure of the p53 R248 hotspot missense mutations that cause human cancer is intended to act as a foundation for improving personalized treatment options for cancer patients. Most frequent missense mutations at the R248 codon of the *p53* gene are associated with the different cancers that were examined in the study. The pathogenicity of the mutations was identified using bioinformatics analyses. We further explored the structural behavior of the wt-p53 and R248 mutant-p53 protein’s binding pattern with the DNA molecule. The precise molecular structure analysis of the p53 highlights the significance of the position R248 for protein stability and functions. The R248W, R248G, and R248P mutants are seen to be deleterious for p53 protein functions. However, our MD simulation data suggest that all five R248 mutations are unstable because they had the least one H-bond interaction with DNA. As a result, the affinity of the p53 protein for DNA binding was decreased. Our study provided insights into the structure and DNA binding affinity of the R248 mutant-p53. It helps to understand the stability, function, dynamics, and regulation and encourages the establishment of p53-targeted anti-cancer treatments. Overall, we believe that the current use of computational technical support will assist the consequence of R248 mutations present in the p53 protein and highlight the potential economic advantage of decreasing the cost of experimental investigations and the time-consuming procedure of mutational assessment.

## 4. Methodology

### 4.1. Retrieval of p53 R248 Mutations in the DNA Binding Residues

We used the COSMIC database (https://cancer.sanger.ac.uk/cosmic, accessed on 30 April 2022) to retrieve the missense mutations of the *p53* gene in DNA binding residue R248. We used the uniport database (https://www.uniprot.org/, accessed on 4 May 2022) to retrieve the protein sequence of p53 in FASTA format [88].

### 4.2. Investigation of Functional Consequences of R248 Mutations

The impartial assessment of eight well-known prediction tools: MAPP, nsSNPAnalyzer, PANTHER, PolyPhen-1, PolyPhen-2, PhD-SNP, SIFT, and SNAP, was conducted using the standard dataset, including approximately 43,000 mutations. The eight finest tools were integrated into a consensus classifier called predict SNP, which considerably increased the prediction performance and made a single reliable tool. We utilized the PredictSNP website to characterize the functional missense mutations.

### 4.3. R248 Mutations Impact p53 Protein Stability Using iStable

Based on the missense mutations wt position and amino acid change, iStable (http://predictor.nchu.edu.tw/iStable/, accessed on 4 May 2022) employed a support vector machine (SVM) to determine the changes in p53 protein stability. The output obtained from iStable integrates many predictors, which include I-Mutant 2.0, MUpro, and iStable, which increases the performance of its meta-prediction rather than a single tool [31].

### 4.4. Biophysical Characterization by Align GVGD

The Align GVGD web-based tool was used to calculate the biophysical characteristics of the missense mutations and predict whether they fall from enriched deleterious to neutral [28]. Align GVGD scores vary from class 0 to class 65, with the following categories: C0, C15, C25, C35, C45, C55, and C65. C0 indicates that the function is least likely to be hindered, while C65 indicates that the function is most likely to be hindered.

### 4.5. Identification of Conserved Regions of p53 Protein Using ConSurf

This server combined two self-governing servers, ConSeq [89] and ConSurf [90]. ConSurf uses the empirical Bayesian inference to examine the evolutionary conservation of P53 protein amino acid substitutions. It displays an evolutionary conservation score from 1 to 9, with the highest value of 9 indicating that the residue is highly conserved. The conservation grades were color-coded onto the surface of the evolutionarily conserved regions of the p53 protein, which were visualized using the Protein explorer engine.

### 4.6. Structural Effects of Missense Mutations Using HOPE

The implications of the mutation on the 3D structure and the accompanying function were hypothesized using the HOPE server. It gathers data from the PDB on protein 3D coordinates, similar to sequence annotations from the DAS prediction and Uniprot database.

### 4.7. Molecular Dynamics Simulation

We retrieved the three-dimensional structure of the p53 protein complexed with DNA (PDB ID: 2AC0 chain A, E and F) holding a resolution of 1.80 Å from the Protein Data bank [91]. Using SwissPDB Viewer, the wild-type p53 protein structure was further introduced with the selected single mutations and saved in PDB format. Before the subsequent analyses, the same software was used to energy minimize the wt and R248 mutant protein [92].

The p53 protein–DNA complexes structure was determined as the input for CHARMM-GUI interoperability to create the wt and five R248 mutant systems (R248W, R248G, R248P, R248L, and R248Q) [93]. The solution builder option in the input generator was used to create each system. Each system was solvated with a TIP3 water box that was rectangular and had a 10 Å distance between its edges. The CHARMM36 all-atom force field produced each system’s topology file and coordinated file [94]. They were all neutral by introducing counter ions to each system in the simulation box. The GROMACS software suite was used to run all the simulations involving the wt DNA complex and the selected mutation complexes of the p53 protein [32]. To eliminate steric overlap, each system underwent 50,000 steps of steepest descent energy reduction [95]. The system was also thoroughly equilibrated before stimulation to reduce unconstrained dynamics. The equilibrium process was split into two parts, namely NVT and NPT. Using the Berendsen algorithm, the standard temperature was kept within the box [96], and the Parrinello–Rahman barostat pressure coupling method was employed during equilibration [97]. For 100 nanoseconds, the MD simulation was performed.

Utilizing GROMACS analysis, the trajectory data were analyzed. Utilizing gmx rmsd, gmx rmsf analysis, RMSD, and RMSF were computed. gmx gyrate was used to compute the radius of gyration in order to ascertain if the system had achieved convergence throughout the 100 ns simulation. Using gmx hbond, the total number of H-bonds formed between the DNA and protein was determined. The solvent-accessible surface areas were calculated using gmx sasa. The MM-PBSA protocols developed in the gmmpbsa package were utilized to compute the binding free energy [98]. To separate biologically important localized motions of protein domains from unimportant localized motions of atoms, a statistical technique known as principal component analysis (PCA) was used to reduce data complexity [99]. For PCA analysis, first, we created the covariance matrix by removing the translational and rotational motions from the system using the gmx covar from GROMACS. The matrix was then diagonalized to determine the eigenvectors and eigenvalues. As they reflect the biggest-amplitude collective movements, the eigenvectors corresponding to the largest eigenvalues are known as “principal components.” Using gmx anaeig analysis, we filtered the original trajectory and projected the portion along the two most significant eigenvectors: vectors 1 and 2. Additionally, using gmx anaeig analysis and a two-dimensional projection, we could see the structural conformations in the subspace along the first two eigenvectors. MATLAB software was used to plot the graphs for all the simulations.

## Figures and Tables

**Table 1:** List of deleterious p53 R248 missense mutations predicted by PredictSNP server.

Mutation	R248W	R248G	R248Q	R248P	R248L
PredictSNP prediction	D	D	D	D	D
MAPP prediction	D	D	D	D	D
PhD-SNP prediction	D	D	D	D	D
PolyPhen-1 prediction	D	D	D	D	D
SIFT prediction	D	D	D	D	D
SNAP prediction	D	D	D	D	D

Note: D, Deleterious.

**Table 2:** The biophysical characterization, evolutionarily conservation, and stability analysis of R248 mutations in p53 protein using Align GVGD, Consurf, and iStable server.

Mutation		R248W	R248G	R248Q	R248P	R248L
Align GVGD		Class C65	Class C65	Class C35	Class C65	Class C65
ConSurf		9	9	9	9	9
iStable	I-Mutant2.0 SEQ	D	D	D	D	D
	DDG	−0.83	−1.74	−1.74	−1.07	−0.83
	MUpro	D	D	D	D	I
	Conf. Score	−0.65126505	−0.89185965	−0.89185965	−0.57518851	0.67336898
	iStable	D	D	D	D	I
	Conf. Score	0.831653	0.859332	0.859332	0.835782	0.506999

Note: D—Decrease; I—Increase.

**Table 3:** Overall binding free energies of DNA to various R248 mutant-p53 protein complexes using MMPBSA calculation, where the binding free energy is mentioned in kJ/mol.

	Wild Type	R248W	R248G	R248L	R248P	R24Q
Van der Waals energy (±SD)	−196.928 +/−63.403	−217.153 +/−31.264	−195.339 +/−37.430	−179.873 +/−22.772	−145.227 +/−44.175	−172.756 +/−32.282
Electrostatic energy (±SD)	−3694.303 +/−774.611	−3507.850 +/−230.814	−2969.158 +/−304.942	−3180.017 +/−199.262	−2917.121 +/−223.878	−3172.238 +/−257.610
Polar solvation energy (±SD)	1013.332 +/−262.915	1191.171 +/−129.089	696.856 +/−189.826	802.547 +/−114.081	801.287 +/−179.410	853.801 +/−141.240
SASA energy (±SD)	−28.858 +/−7.108	−31.040 +/−4.048	−24.867 +/−4.462	−26.327 +/−3.180	−22.537 +/−4.612	−25.422 +/−3.371
Total Binding energy (±SD)	−2906.756 +/−621.185	−2564.871 +/−194.196	−2492.508 +/−215.762	−2583.670 +/−186.701	−2283.598 +/−169.333	−2516.614 +/−227.002

**Table 4:** Conventional H-bonds formed between the p53 protein amino acid Arg248 and DNA in different mutations.

	No. of H-Binding Site at Arg248	Conventional H-Binding of Arg248 with DNA
WT	3	A:Arg248:NH2-E:Gua12:O2P
		A:Arg248:NH2-F:Ade6:O3′
		A:Arg248:NE-F:Thy7:O1P
R248W	1	A:TRP248:HD1-E:THY7:O1P
R248G	0	Nil
R248L	0	Nil
R248P	0	Nil
R24Q	1	A: GLN248:HE21-E:THY7:O1P

Note: A—A chain of p53 protein; E—E chain of DNA; F—F chain of DNA.

## Data Availability

Not applicable.
